# A Simple Scoring Model for Prediction of Rupture Risk of Anterior Communicating Artery Aneurysms

**DOI:** 10.3389/fneur.2019.00520

**Published:** 2019-05-31

**Authors:** Guang-xian Wang, Shuang Wang, Lan-lan Liu, Ming-fu Gong, Dong Zhang, Chun-yang Yang, Li Wen

**Affiliations:** Department of Radiology, Xinqiao Hospital, Third Military Medical University, Chongqing, China

**Keywords:** CT angiography, anterior communicating artery aneurysms, risk factors, patient characteristics, morphological parameter, predictive scoring model

## Abstract

**Background:** The rupture risk of anterior communicating artery aneurysms (ACoAAs) has been known to be higher than that of aneurysms at other locations. Thus, the aim of this study is to investigate the clinical and morphological characteristics associated with risk factors for the rupture of ACoAAs.

**Methods:** In total, 361 consecutive patients with 361 ACoAAs between August 2011 and December 2017 were retrospectively reviewed. Patients and ACoAAs were divided into ruptured and unruptured groups. In addition to clinical characteristics, ACoAA characteristics were evaluated by CT angiography (CTA). A multiple logistic regression analysis was used to identify the independent risk factors associated with ACoAA rupture. The assignment score of these variables depends on the β coefficient. A receiver operating characteristic (ROC) curve analysis was used to calculate the optimal thresholds.

**Results:** The multiple logistic regression model revealed that A1 dominance [odds ratio (OR) 3.034], an irregular shape (OR 3.358), and an aspect ratio ≥1.19 (AR; OR 3.163) increased the risk of rupture, while cerebral atherosclerosis (OR 0.080), and mean diameters ≥2.48 mm (OR 0.474) were negatively correlated with ACoAA rupture. Incorporating these five factors, the ROC analysis revealed that the threshold value of the multifactors was one, the sensitivity was 88.3%, and the specificity was 66.0%.

**Conclusions:** The scoring model is a simple method that is based on A1 dominance, irregular shape, aspect ratio, cerebral atherosclerosis, and mean diameters from CTA and is of great value in the prediction of the rupture risk of ACoAAs.

## Introduction

Intracranial aneurysms (IAs) are common, and their ruptures are one of the main causes of subarachnoid hemorrhage (SAH). In clinical practice, the treatment decisions related to incidental unruptured IAs (UIAs) still need careful consideration because most UIAs do not rupture during patients' lifetimes, and prophylactic treatment is also associated with risks. Thus, the assessment of the rupture risk for UIAs has important clinical value.

Although many previous studies showed that the locations of UIAs were not associated with an increased rupture risk after a long period of follow-up (1, 2), the American Heart Association/American Stroke Association indicated that the treatment decision regarding UIAs is based mainly on the size and location (3). However, the influence of aneurysm size does not seem to be the same for all locations (4). Some studies showed that the risk factors of UIA ruptures differ by their location (5–7). Therefore, the natural history of UIAs may be individually investigated for each distinct location.

Anterior communicating artery aneurysms (ACoAAs) account for ~30–37% of all IAs, and the rupture rate of ACoAAs is the highest compared with other types (4, 8–10). In addition, the proximity of ACoAAs to important midline structures, especially the optic apparatus, leads to high morbidity and mortality rates following rupture (8). Thus, it is necessary to assess the risk for rupture of ACoAAs and then implement effective treatments to prevent such severe consequences. However, only a few publications have focused on the patient and morphological risk factors for the rupture of ACoAAs (9–15), and the results are not consistent. To our knowledge, there is no simple risk score for ACoAA ruptures only. An effective quantitative scoring model based on patients' characteristics and ACoAA characteristics for ACoAA rupture would be of enormous clinical value. The aim of this study is to establish a simple scoring model for the prediction of rupture risk of ACoAAs.

## Materials and Methods

### Patients

This retrospective study was approved by our institutional ethics committee. From August 2011 to December 2017, a total of 423 consecutive patients underwent head computed tomography angiography (CTA) examinations and were diagnosed with anterior cerebral artery aneurysms. Aneurysms arising from the A1–A2 junction were selected, excluding A1 segment aneurysms (*n* = 21) or distal anterior cerebral artery aneurysms (*n* = 22). Mycotic aneurysms (*n* = 1), cases associated with arteriovenous malformations (*n* = 6), and aneurysms with poor image quality (*n* = 12) were not eligible for this study. All ruptured intracranial aneurysms (RIAs) were managed with treatment (coiling or clipping), 95 UIAs were managed because of clinical symptoms (e.g., headache), and 54 UIAs were observed. Between follow-up with CTA or magnetic resonance angiography (MRA), six aneurysms grew and two ruptured, and the ruptured aneurysms were sorted into the ruptured group. Finally, 361 patients (214 in the ruptured group and 147 in the unruptured group) were available for the analysis.

Patients' clinical data were extracted from the hospital's medical records by one of the assessors. A history of hypertension, heart disease, diabetes mellitus, cerebral atherosclerosis (CA), alcohol consumption, cigarette smoking, and SAH was recorded as either present or absent. A history of SAH was defined as SAH due to the rupture of an aneurysm in other locations. In cases with multiple IAs, the RIAs were determined based on the CT, angiographic, or operative findings.

### Computed Tomography Angiography and Image Analysis

All CTAs were performed on a 64-slice CT machine (GE LightSpeed VCT; GE Healthcare, WI, USA). The CTA images were evaluated on the GE Advantix workstation (Advantage Windows 4.5). Then, the three-dimensional (3D) volume rendering (VR) and maximum intensity projection (MIP) were obtained. All morphological variables were independently obtained by two observers (one of the observers has 18 years of experience in neuroradiology, and the other has 8 years of experience in vascular imaging), and the average value was used for subsequent statistical analyses. Controversial cases were resolved through discussion.

**Figure 1:**
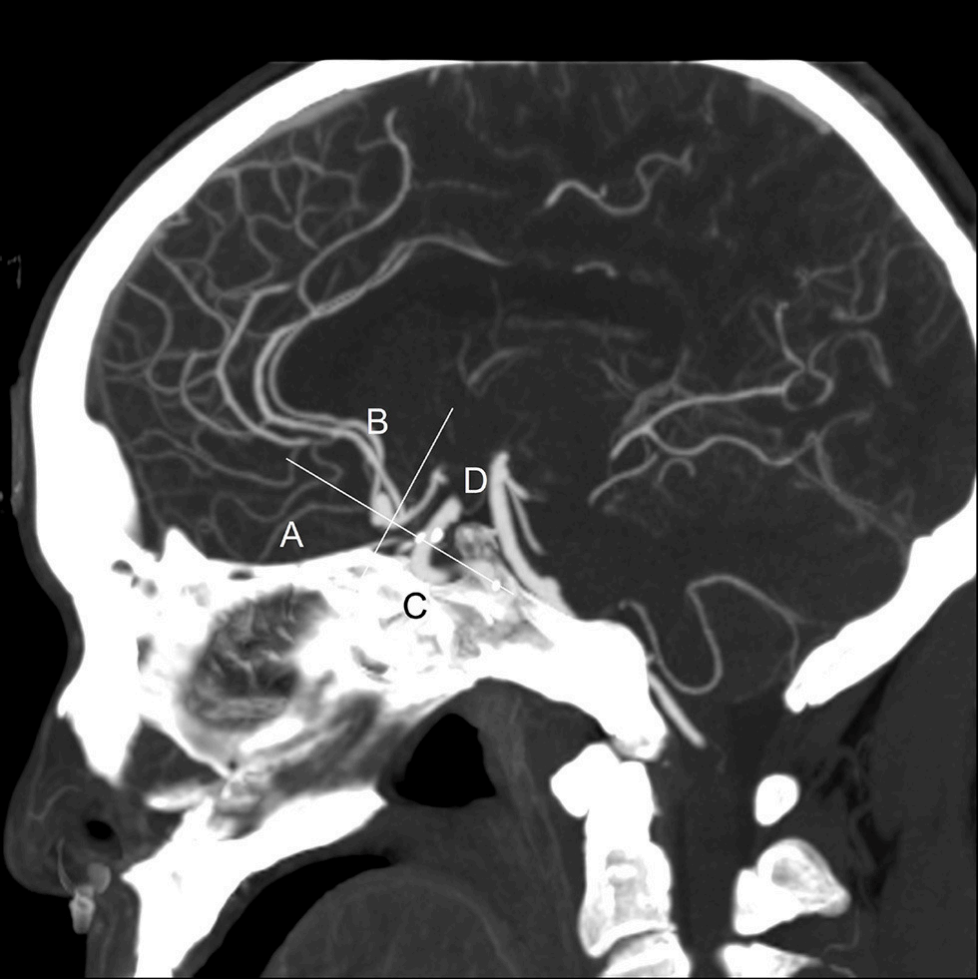
This is a sagittal maximum intensity projection (MIP) of a computed tomography angiography (CTA) from a typical patient. The directions of aneurysm dome were defined as anterior–inferior (A), anterior–superior (B), posterior–inferior (C), and posterior–superior (D). This picture depicts the direction of the aneurysm dome as B.

**Figure 2:**
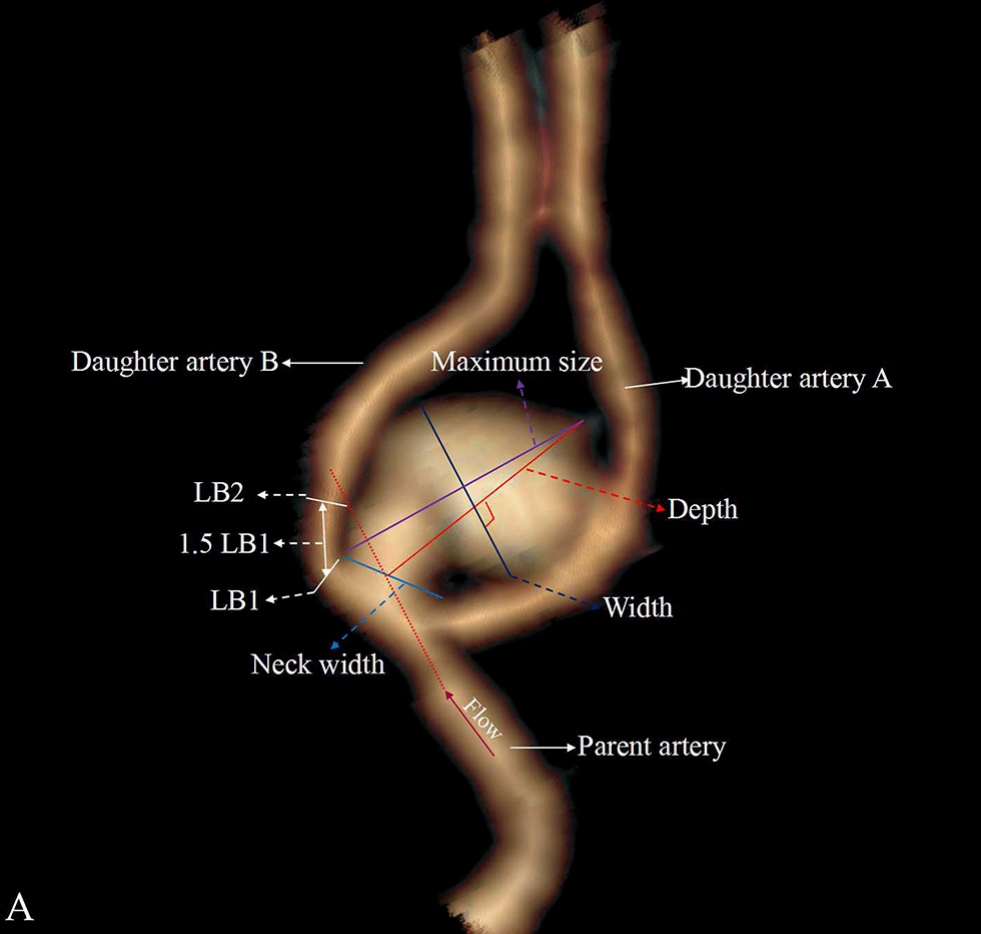
Aneurysm with A1 dominance and is classified as a deviated neck type (type D). The larger daughter artery is defined as daughter artery A, and the small daughter artery is defined as daughter artery B. LB mean diameter of the daughter artery B is measured at LB1 and LB2, and the daughter artery A and parent artery are measured as LA and LP. The mean diameter is defined as (LA + LB + LP)/3.

**Figure 3:**
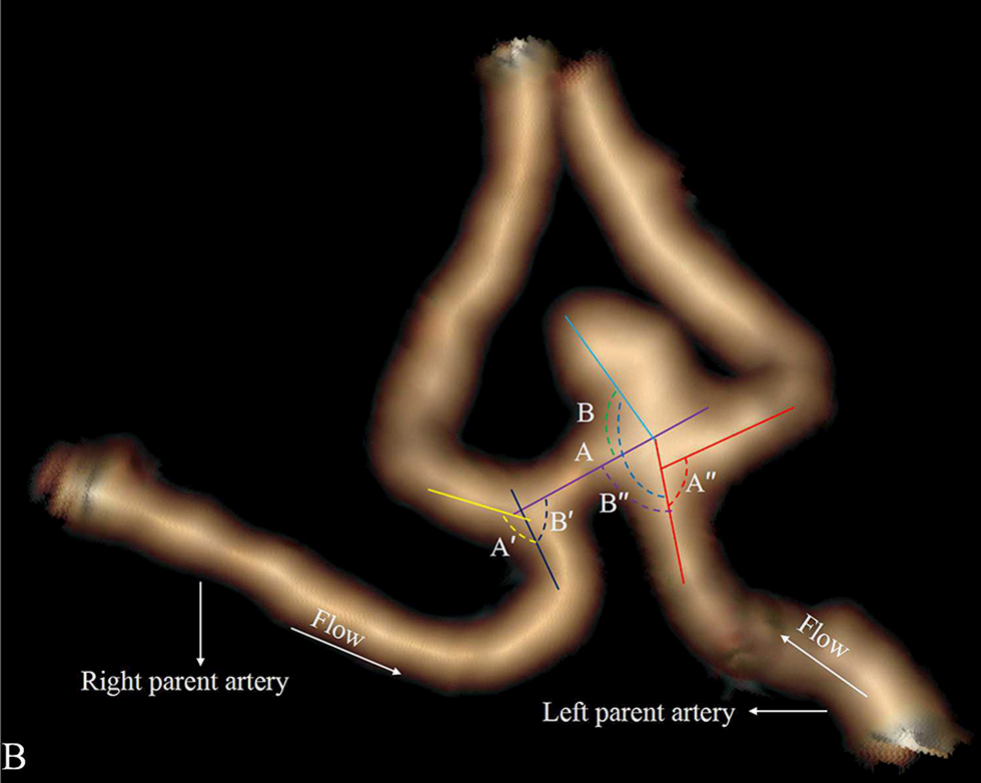
Aneurysm with A1 co-dominance. The LAR is defined as (angle A″/angle B″+angle A′/angle B′)/2. Flow angle (FA) is defined as between the vector of depth of the aneurysm and the vector of the centerline of the parent artery. FA is defined as (flow angle A + flow angle B)/2.

Four categorical morphological variables included the shape of the aneurysm (simple-lobed and irregularly shaped aneurysms), the variation in the A1 segment, the neck types, and the direction of the aneurysm dome. An aneurysm with an irregular shape was defined as having lobular or daughter sacs (16, 17). A1 co-dominance was defined as a < 33% difference in diameter between the two A1 segments; A1 dominance was defined as a >33% difference in diameter between the two A1 segments (9, 10, 12). Based on the available literature (9, 10, 12), the aneurysm dome was classified based on four directions: anterior–inferior, anterior–superior, posterior–inferior, and posterior–superior, called A, B, C, and D, respectively (Figure 1). Based on the neck location, the neck types were classified as type C or type D (16, 17). Seven continuous morphological variables, including the aneurysm depth (the longest diameter between the neck and the dome), the width (the maximum distance perpendicular to the depth), the neck width (the largest cross-sectional diameter of the aneurysm neck), the maximum size (Dmax, the largest measurement in terms of maximum dome diameter or width), the mean diameters of the parent and two daughter arteries (MD), the flow angle (FA), and the parent–daughter angle, were evaluated (Figure 2). Five secondary geometric indices, including the aspect ratio (AR; depth/neck width), the depth–width ratio (DW; depth/width), the bottleneck factor (BF; width/neck width), the size ratio (SR; depth/MD), and the lateral angle ratio (LAR; parent–larger daughter angle/parent–smaller daughter angle), were calculated. These variables have already been used and are clearly defined in the literature (9–18). Of note, SR, FA, parent–daughter angle, and LAR were measured differently based on whether or not A1 co-dominance was present (Figure 3).

### Statistical Analysis

All statistics were performed using SPSS 17.0 (SPSS Inc., Chicago, IL, USA), and *P* ≤ 0.05 was considered statistically significant. The agreement between two observers for the shape of the IAs, A1 segment, the neck types, and the direction of the aneurysm dome was evaluated by a kappa value. All data were expressed as the means ± standard deviations or the number of patients or aneurysms (%). Continuous data were compared using a two-tailed independent Student's *t*-test (for normally distributed data) or the Mann–Whitney *U*-test (for non-normally distributed data); categorical data were compared using a chi-square test. All variables (*P* ≤ 0.2) were entered into the univariate analysis. A conditional multiple logistic regression analysis with forward selection was used to calculate the independent risk factors associated with ACoAA rupture for those features that achieved statistical significance (*P or* 0.05) in the univariate analysis. The assignment score of these independent risk factors depends on the β coefficient in the multiple logistic regression model. A receiver operating characteristic (ROC) curve analysis was performed to determine the cutoff value, the sensitivity, and the specificity.

## Results

The data of all the patients and aneurysms are showed as Data Sheet 1. The clinical characteristics of the 361 patients are listed in Table 1. The mean age was 56.63 ± 9.85 years: 53.87 ± 9.18 years for the ruptured group and 60.65 ± 9.63 years for the unruptured group. The patients' age, heart disease, CA, and SAH history were correlated with the risk of ACoAA rupture.

**Table 1:** Clinical characteristics with ruptured and unruptured aneurysms.

**Clinical data**	**Patient groups**		***P***
	**Unruptured (*n* = 147)**	**Ruptured (*n* = 214)**	
Male	83 (56.5%)	116 (54.2%)	0.747
Age^*^	60.65 ± 9.63	53.87 ± 9.18	< 0.001
Hypertension	85 (57.8%)	67 (31.3%)	0.280
Heart disease^*^	21 (14.3%)	5 (2.3%)	< 0.001
Diabetes mellitus	10 (6.8%)	8 (3.7%)	0.222
Cerebral atherosclerotic^*^	49 (33.3%)	8 (3.7%)	< 0.001
Alcohol history	44 (29.9%)	68 (31.8%)	0.730
Cigarette smoking	55 (37.4%)	81 (37.9%)	1.000
SAH history	11 (7.5%)	7 (3.3%)	0.086
Multiple aneurysms	13 (8.8%)	12 (5.6%)	0.292

SAH, subarachnoid hemorrhage.

* *Variables showing significant difference by univariate analysis (P < 0.05)*.

Inter-observer agreement on the CTA categorical factors was good (κ = 0.939 for the shape of the IAs, κ = 0.966 for the A1 segment, κ = 1.000 for the neck types, and κ = 0.959 for the direction of the aneurysm dome). Table 2 shows the morphological characteristics of the ACoAAs. Irregular shape, A1 dominance, depth, width, Dmax, AR, DW, BF, SR, MD, and parent–smaller daughter angle were associated with rupture risk.

**Table 2:** The morphological characteristics with ruptured and unruptured aneurysms.

**Morphologic parameters**	**Aneurysm groups**		***P***
	**Unruptured (*n* = 147)**	**Ruptured (*n* = 214)**	
Irregular shape^*^	46 (31.3%)	151 (70.6%)	< 0.001
Type C	93 (63.3%)	144 (67.3%)	0.432
A1 dominance^*^	105 (71.4%)	183 (85.5%)	0.044
**Direction of dome**			
A	54 (36.7%)	80 (37.4%)	0.912
B	25 (17.0%)	28 (13.1%)	0.364
C	55 (37.4%)	84 (39.3%)	0.742
D	13 (8.8%)	22 (10.3%)	0.720
Neck width (mm)	4.06 ± 1.58	3.84 ± 1.31	0.406
Width (mm)^*^	4.17 ± 2.26	5.03 ± 2.46	< 0.001
Depth (mm)^*^	4.37 ± 2.51	5.87 ± 2.49	< 0.001
Maximum diameter (mm)^*^	5.27 ± 2.62	6.88 ± 2.78	< 0.001
AR^*^	1.09 ± 0.53	1.59 ± 0.69	< 0.001
DW^*^	1.06 ± 0.38	1.24 ± 0.38	< 0.001
BF^*^	1.03 ± 0.37	1.33 ± 0.52	< 0.001
MD (mm)^*^	2.55 ± 0.45	2.32 ± 0.44	< 0.001
SR^*^	1.78 ± 1.08	2.69 ± 1.36	< 0.001
Flow angle (°)	131.43 ± 22.82	130.57 ± 20.78	0.625
Parent–larger daughter angle (°)	98.22 ± 23.0	101.87 ± 23.92	0.123
Parent–smaller daughter angle (°)^*^	95.03 ± 22.52	103.32 ± 23.68	0.002
LAR	1.10 ± 0.42	1.04 ± 0.38	0.306

A, anterior–inferior; B, anterior–superior; C, posterior–inferior; D, posterior–superior; AR, aspect ratio; DW, depth/width ratio; BF, bottleneck factor; MD, mean diameter; SR, size ratio; FR, flow angle; LAR, lateral angle ratio.

* *Variables showing significant difference by univariate analysis (P < 0.05)*.

Fourteen independent variables (*P* ≤ 0.05) were used in the multiple logistic regression analysis. The model showed that five variables were associated with the rupture risk of ACoAAs, among which A1 dominance, an irregular shape, and a large AR strongly increased the rupture risk, whereas CA and MD decreased the rupture risk (Table 3), with odds ratios (ORs) of 3.034, 3.358, 3.163, 0.080, and 0.474, respectively. The threshold values of AR and MD were 1.19 and 2.48 mm, respectively.

**Table 3:** Multiple logistic regression analysis for the prediction of aneurysm rupture.

**Variable**	**Odds ratio**	***P***	**95% CI**	**β**
Cerebral atherosclerotic	0.080	< 0.001	0.033–0.190	−2.529
A1 dominance	3.034	0.001	1.580–5.827	1.110
Irregular shape	3.358	< 0.001	1.917–5.880	1.211
Aspect ratio	3.163	< 0.001	1.742–5.745	1.152
Mean diameter	0.474	0.02	0.253–0.890	−0.746

*CI, confidence intervals; β, partial regression coefficient*.

According to the β coefficient, we established a predictive scoring model for ACoAA–rupture risk, and points were assigned as follows. For the presence of A1 dominance, irregular shape, and an AR ≥ 1.19 (β = 1.110, 1.211, and 1.152, respectively), the score = 1; if there was A1 co-dominance, regular shape, and an AR < 1.19, then the score = 0. For the presence of CA (β = −2.529), score = −2; otherwise, the score = 0. For an MD ≥ 2.48 mm, the score = −0.5 (β = −0.746). For an MD < 2.48 mm, the score = 0.5. The optimal cutoff value of the predictive score was 1 on the basis of the maximum Youden's index, and the value of the area under the curve was 0.846; the sensitivity and specificity for the detection of RIAs were 88.3 and 66.0%, respectively (Table 4). The incidence of RIAs in the low-risk group (score < 1) and the high-risk group (score ≥ 1) was 20.5 and 79.1%, respectively (Table 5).

**Table 4:** Area under the curve for predictive score.

**Factor**	**Area**	**Threshold value**	***P***	**Sen (%)**	**Spe (%)**	**95% CI**
Predictive score	0.846	1	< 0.001	88.3	66.0	0.806–0.887

*Sen, sensitivity; Spe, specificity; CI, confidence intervals*.

**Table 5:** Incidence of aneurysm rupture based on the predictive scoring model.

**Score**	**Total (*n*)**	**RIAs (*n*)**	**Risk**	**Incidence of RIAs**
−2.5	6	0	Low	20.5%
−1.5	17	1		
−0.5	22	1		
0.5	77	23		
1.5	72	45	High	79.1%
2	1	1		
2.5	87	69		
3.5	79	74		

*RIAs, ruptured intracranial aneurysms*.

## Discussion

The rupture rate for aneurysms in the ACoAA is higher than those in the other sites (8–10), and this study indicated that the rupture rate of ACoAAs was 59.3% (214/361). In addition, ACoAAs are proximal to important midline structures, especially the optic apparatus, leading to high morbidity and mortality rates following rupture (8). The bifurcation areas of arteries are known to be vulnerable sites where the wall is weak and the hemodynamic stress changes (16, 17). Moreover, hemodynamics are strongly dependent on the feeding vessel (19), so the hemodynamics of ACoAAs may be different from that of anterior cerebral artery aneurysms. Therefore, in this study, we excluded proximal and distal IAs to identify risk factors related to ACoAA rupture. Our results showed that MD and CA were negatively associated with rupture, while ACoAAs with A1 dominance, an irregular shape, and a high AR were more prone to rupture.

The clinical characteristics are believed to be important factors related to aneurysm rupture. Age is known to be a significant risk factor for the rupture of IAs, and many previous studies showed that patients with RIAs were younger than those with UIAs (6, 10, 16, 17). Although most studies showed that patients with ruptured ACoAAs were younger than those with UIAs (9–11, 13–15), this factor was not significant upon multiple analysis. The incidence of CA increased with age. It is well-known that CA is more prevalent in Asian people than in people from Western countries, and atherosclerotic or calcified walls decrease the risk of IA rupture (20). This study and our previous studies showed that CA decreased the risk of IA rupture (16, 17), possibly because CA or calcified walls slow the flow rates entering the aneurysm and thus reduce its wall shear stress (21).

The formation of ACoAAs is more likely when there is an A1 segment predominance in the anterior cerebral artery (22). In this study, ACoAAs were most commonly found on the A1 dominant side. Previous studies showed that A1 dominance was not associated with ruptured ACoAAs (9–12, 15, 22). In contrast, our study demonstrated that A1 dominance was closely related to ruptured ACoAAs, which agreed with the results reported by Xu et al. (13) and Kim and Hwang (14), possibly because the A1 dominant segment has more blood flow stress and then gives rise to a greater risk of rupture (13).

An aneurysm with daughter sacs or a lobular shape was defined as having an irregular shape (16–18). Most, but not all, previous retrospective studies showed that IAs with irregular shapes were associated with a higher risk of rupture (10, 12, 16–18) and the rupture risk increased according to the extent of morphologic changes (23). Additionally, a prospective study also indicated that UIAs with daughter sacs were also more likely to rupture (24). Although some studies showed different results (9, 11, 13–15), the present results showed that irregular aneurysms are more likely to rupture, which is in accordance with previous ACoAA studies (10, 12). Possible reasons for this result are that aneurysm wall irregularity accelerates histological wall degeneration, daughter sac walls are thinner than other sites, and the irregular shape leads to blood flow pattern instabilities (18, 25).

Many previous studies on AR and SR produced conflicting results. Most groups have reported that SR rather than AR is a risk factor for ACoAA rupture (9, 11, 13, 15). However, other researchers have shown that a large AR increases the risk of ACoAA rupture (12, 14). These conflicting results may be due to the use of different imaging modalities and measurement methodologies. The present data showed that AR strongly increased the risk of ACoAA rupture, and the threshold value was 1.19, which was concordant with our previous reports (16).

In this study, we found that MD was associated with a decreased risk of ACoAA rupture, indicating that a smaller artery is associated with a higher risk of rupture. This result also agreed with our previous studies (17, 18), possibly because IAs arising from smaller vessels have thinner walls and would experience greater wall tension than larger vessels (17, 18).

Based on the five factors, we established a simple scoring model for predicting the rupture risk of ACoAAs. Using one as the cutoff value, the sensitivity and specificity values for the detection of RIAs were 88.3 and 66.0%, respectively, and the positive and negative predictive values were 79.1 and 79.5%, respectively. However, some ACoAAs with threshold values smaller than one still ruptured; one reason for this finding is that CA lowers the score; another reason for this finding is that patients' clinical factors, such as family history and congenital diseases, were not used in this study because these data were not recorded for many of the older patients, but these clinical factors may cause the rupture of IAs (26). This scoring model differs from the PHASES score, which was based on prospective data from six cohort studies on rupture risk of unruptured IAs and reported that patients' geographic location, age, hypertension status, previous SAH from a different IA, IA size, and location were associated with IA rupture (5). Moreover, different imaging modalities were used to assess the initial IA characteristics, and rupture risk factors such as IA shape could not be included in the PHASES score. Finally, a potential selection bias exists in the PHASES score because many patients were removed from those studies when they received treatment before IA rupture.

## Limitations

The present study has several limitations. First, this is a retrospective analysis of patients with a small sample size of ACoAAs from one hospital, and patients' confounding clinical characteristics may lead to statistical bias. Second, the ACoAAs were not monitored in real time, and the shape or size of the ruptured ACoAAs might have changed due to the rupture, and the results may be biased, even though previous studies reported that aneurysms do not shrink in size after rupture (27, 28). Third, the unruptured ACoAAs may rupture in the future. It is ideal to dynamically observe the size and morphological changes. Fourth, our study has referral bias because it was performed in a cerebral surgery center in which a high proportion of patients exhibited a rupture. In the future, a multicenter, prospective study with a large sample size is needed.

## Conclusion

We found that MD and CA are likely protective factors against ACoAA rupture. However, morphological characteristics such as A1 dominance, aneurysm with an irregular shape, and a high AR (>1.19) are risk factors for rupture. The predictive scoring model according to the five factors is of great value in predicting the rupture risk of ACoAAs, and more attention should be paid to the aneurysms when the score ≥ 1 during clinical practice. By applying this scoring model, clinicians can predict which patients with ACoAAs may have a high risk of rupture and thus help patients make the right decisions.

## Data Availability

Publicly available datasets were analyzed in this study. This data can be found here: “https://www.frontiersin.org/submission/submit?status=p&amp;id=346483.”

## Ethics Statement

This retrospective study was approved by the institutional ethics committee of Xinqiao hospital, 2016031.

## Author Contributions

LW: conceptualization. SW: data curation. LL, MG, DZ, and CY: investigation. LW: methodology. GW: writing—original draft.

### Conflict of Interest Statement

The authors declare that the research was conducted in the absence of any commercial or financial relationships that could be construed as a potential conflict of interest.

## References

[1] Villablanca JP Duckwiler GR Jahan R Tateshima S Martin NA Frazee J. Natural history of asymptomatic unruptured cerebral aneurysms evaluated at CT angiography: growth and rupture incidence and correlation with epidemiologic risk factors. Radiology. (2013) 269:258–65. 10.1148/radiol.13121188 23821755

[2] Mehan WA Jr Romero JM Hirsch JA Sabbag DJ Gonzalez RG Heit JJ. Unruptured intracranial aneurysms conservatively followed with serial CT angiography: could morphology and growth predict rupture? J Neurointerv Surg. (2014) 6:761–6. 10.1136/neurintsurg-2013-010944 24275611

[3] Thompson BG Brown RD Jr Amin-Hanjani S Broderick JP Cockroft KM Connolly ES Jr. Guidelines for the management of patients with unruptured intracranial aneurysms: a guideline for healthcare professionals from the American Heart Association/American Stroke Association. Stroke. (2015) 46:2368–400. 10.1161/STR.0000000000000070 26089327

[4] Mira JM Costa FA Horta BL Fabiao OM. Risk of rupture in unruptured anterior communicating artery aneurysms: meta-analysis of natural history studies. Surg Neurol. (2006) 66:S12–9. 10.1016/j.surneu.2006.06.025 17081844

[5] Greving JP Wermer MJ Brown RD Jr Morita A Juvela S Yonekura M. Development of the phases score for prediction of risk of rupture of intracranial aneurysms: a pooled analysis of six prospective cohort studies. Lancet Neurol. (2014) 13:59–66. 10.1016/S1474-4422(13)70263-1 24290159

[6] Heit JJ Gonzalez RG Sabbag D Brouwers HB Ordonez Rubiano EG Schaefer PW. Detection and characterization of intracranial aneurysms: a 10-year multidetector CT angiography experience in a large center. J Neurointerv Surg. (2016) 8:1168–72. 10.1136/neurintsurg-2015-012082 26553878

[7] Kang H Ji W Qian Z Li Y Jiang C Wu Z. Aneurysm characteristics associated with the rupture risk of intracranial aneurysms: a self-controlled study. PLoS ONE. (2015) 10:e0142330. 10.1371/journal.pone.0142330 26540158 PMC4634979

[8] Agrawal A Kato Y Chen L Karagiozov K Yoneda M Imizu S. Anterior communicating artery aneurysms: an overview. Minim Invasive Neurosurg. (2008) 51:131–5. 10.1055/s-2008-1073169 18521782

[9] Cai W Shi D Gong J Chen G Qiao F Dou X. Are morphologic parameters actually correlated with the rupture status of anterior communicating artery aneurysms? World Neurosurg. (2015) 84:1278–83. 10.1016/j.wneu.2015.05.060 26054869

[10] Matsukawa H Uemura A Fujii M Kamo M Takahashi O Sumiyoshi S. Morphological and clinical risk factors for the rupture of anterior communicating artery aneurysms. J Neurosurg. (2013) 118:978–83. 10.3171/2012.11.JNS121210 23240701

[11] Lin N Ho A Charoenvimolphan N Frerichs KU Day AL Du R. Analysis of morphological parameters to differentiate rupture status in anterior communicating artery aneurysms. PLoS ONE. (2013) 8:e79635. 10.1371/journal.pone.0079635 24236149 PMC3827376

[12] Choi JH Jo KI Kim KH Jeon P Yeon JY Kim JS. Morphological risk factors for the rupture of anterior communicating artery aneurysms: the significance of fenestration. Neuroradiology. (2016) 58:155–60. 10.1007/s00234-015-1610-9 26511858

[13] Xu T Lin B Liu S Shao X Xia N Zhang Y. Larger size ratio associated with the rupture of very small ( ≤ 3 mm) anterior communicating artery aneurysms. J Neurointerv Surg. (2017) 9:278–82. 10.1136/neurintsurg-2016-012294 27009240

[14] Kim MC Hwang SK. The rupture risk of aneurysm in the anterior communicating artery: a single center study. J Cerebrovasc Endovasc Neurosurg. (2017) 19:36–43. 10.7461/jcen.2017.19.1.36 28503486 PMC5426194

[15] Shao X Wang H Wang Y Xu T Huang Y Wang J. The effect of anterior projection of aneurysm dome on the rupture of anterior communicating artery aneurysms compared with posterior projection. Clin Neurol Neurosurg. (2016) 143:99–103. 10.1016/j.clineuro.2016.02.023 26914141

[16] Wang G-X Zhang D Wang Z-P Yang L-Q Zhang L Wen L. Risk factors for the rupture of bifurcation intracranial aneurysms using ct angiography. Yonsei Med J. (2016) 57:1178–84. 10.3349/ymj.2016.57.5.1178 27401649 PMC4960384

[17] Wang GX Yu JY Wen L Zhang L Mou KJ Zhang D. Risk factors for the rupture of middle cerebral artery bifurcation aneurysms using CT angiography. PLoS ONE. (2016) 11:e0166654. 10.1371/journal.pone.0166654 27977691 PMC5157982

[18] Wang GX Wen L Yang L Zhang QC Yin JB Duan CM Risk factors for the rupture of intracranial aneurysms using CT angiography. World Neurosurg. (2018) 110:e333–8. 10.1016/j.wneu.2017.10.174 29129762

[19] Lu G Huang L Zhang XL Wang SZ Hong Y Hu Z. Influence of hemodynamic factors on rupture of intracranial aneurysms: patient-specific 3d mirror aneurysms model computational fluid dynamics simulation. AJNR Am J Neuroradiol. (2011) 32:1255–61. 10.3174/ajnr.A2461 21757526 PMC7966033

[20] Wermer MJ van der Schaaf IC Algra A Rinkel GJ. Risk of rupture of unruptured intracranial aneurysms in relation to patient and aneurysm characteristics: an updated meta-analysis. Stroke. (2007) 38:1404–10. 10.1161/01.STR.0000260955.51401.cd 17332442

[21] Nahed BV DiLuna ML Morgan T Ocal E Hawkins AA Ozduman K. Hypertension, age, and location predict rupture of small intracranial aneurysms. Neurosurgery. (2005) 57:676–83. 10.1227/01.NEU.0000175549.96530.59 16239879

[22] Ye J Zheng P Hassan M Jiang S Zheng J. Relationship of the angle between the A1 and A2 segments of the anterior cerebral artery with formation and rupture of anterior communicating artery aneurysm. J Neurol Sci. (2017) 375:170–4. 10.1016/j.jns.2017.01.062 28320123

[23] Abboud T Rustom J Bester M Czorlich P Vittorazzi E Pinnschmidt HO. Morphology of ruptured and unruptured intracranial aneurysms. World Neurosurg. (2017) 99:610–7. 10.1016/j.wneu.2016.12.053 28017741

[24] Japan Investigators UCAS Morita A Kirino T Hashi K Aoki N Fukuhara S. The natural course of unruptured cerebral aneurysms in a Japanese cohort. N Engl J Med. (2012) 366:2474–82. 10.1056/NEJMoa1113260 22738097

[25] Huhtakangas J Lehecka M Lehto H Jahromi BR Niemelä M Kivisaari R. CTA analysis and assessment of morphological factors related to rupture in 413 posterior communicating artery aneurysms. Acta Neurochir. (2017) 159:1643–52. 10.1007/s00701-017-3263-4 28710522

[26] Bacigaluppi S Piccinelli M Antiga L Veneziani A Passerini T Rampini P. Factors affecting formation and rupture of intracranial saccular aneurysms. Neurosurg Rev. (2014) 37:1–14. 10.1007/s10143-013-0501-y 24306170

[27] Rahman M Ogilvy CS Zipfel GJ Derdeyn CP Siddiqui AH Bulsara KR. Unruptured cerebral aneurysms do not shrink when they rupture: multicenter collaborative aneurysm study group. Neurosurgery. (2011) 68:155–60. 10.1227/NEU.0b013e3181ff357c 21150760

[28] Yi J Zielinski D Chen M. Cerebral aneurysm size before and after rupture: case series and literature review. J Stroke Cerebrovasc Dis. (2016) 25:1244–8. 10.1016/j.jstrokecerebrovasdis.2016.01.031 26935121

